# The respiratory physiome: Clustering based on a comprehensive lung function assessment in patients with COPD

**DOI:** 10.1371/journal.pone.0201593

**Published:** 2018-09-12

**Authors:** Ingrid M. L. Augustin, Martijn A. Spruit, Sarah Houben-Wilke, Frits M. E. Franssen, Lowie E. G. W. Vanfleteren, Swetlana Gaffron, Daisy J. A. Janssen, Emiel F. M. Wouters

**Affiliations:** 1 CIRO+, center of expertise for chronic organ failure, Horn, The Netherlands; 2 Department of Respiratory Medicine, Maastricht University Medical Centre+ (MUMC+), Maastricht, the Netherlands; 3 Viscovery Software GmbH, Vienna, Austria; National and Kapodistrian University of Athens, GREECE

## Abstract

**Background:**

While spirometry and particularly airflow limitation is still considered as an important tool in therapeutic decision making, it poorly reflects the heterogeneity of respiratory impairment in chronic obstructive pulmonary disease (COPD). The aims of this study were to identify pathophysiological clusters in COPD based on an integrated set of standard lung function attributes and to investigate whether these clusters can predict patient-related outcomes and differ in clinical characteristics.

**Methods:**

Clinically stable COPD patients referred for pulmonary rehabilitation underwent an integrated assessment including clinical characteristics, dyspnea score, exercise performance, mood and health status, and lung function measurements (post-bronchodilator spirometry, body plethysmography, diffusing capacity, mouth pressures and arterial blood gases). Self-organizing maps were used to generate lung function based clusters.

**Results:**

Clustering of lung function attributes of 518 patients with mild to very severe COPD identified seven different lung function clusters. Cluster 1 includes patients with better lung function attributes compared to the other clusters. Airflow limitation is attenuated in clusters 1 to 4 but more pronounced in clusters 5 to 7. Static hyperinflation is more dominant in clusters 5 to 7. A different pattern occurs for carbon monoxide diffusing capacity, mouth pressures and for arterial blood gases. Related to the different lung function profiles, clusters 1 and 4 demonstrate the best functional performance and health status while this is worst for clusters 6 and 7. All clusters show differences in dyspnea score, proportion of men/women, age, number of exacerbations and hospitalizations, proportion of patients using long-term oxygen and number of comorbidities.

**Conclusion:**

Based on an integrated assessment of lung function variables, seven pathophysiological clusters can be identified in COPD patients. These clusters poorly predict functional performance and health status.

## Introduction

Chronic obstructive pulmonary disease (COPD) is a common, preventable and treatable disease that is characterized by persistent respiratory symptoms and airflow limitation that is due to airway and/or alveolar abnormalities usually caused by significant exposure to noxious particles or gases [1]. While it is widely recognized that COPD is a complex, heterogeneous disease with pulmonary and extra-pulmonary manifestations [2], post-bronchodilator spirometry remains the diagnostic test to diagnose the disease, classify the degree of airflow limitation [1], monitor disease progression [3] and response to pharmacotherapies [4]. Nevertheless, the degree of airflow limitation correlates only moderately to exercise performance, symptom burden, mood and health status in patients with COPD [5–7].

Pathophysiology of COPD is far more complex than just airflow limitation. Indeed, lung hyperinflation is one of the hallmarks of patients with COPD [8]. Lungs can be hyperinflated at rest (static hyperinflation) and/or during exercise (dynamic hyperinflation) [9]. Lung hyperinflation can affect respiratory muscle function in patients with COPD [10]. Impaired diffusing capacity of the lung is another characteristic in a subgroup of patients with COPD [8]; when there is a loss of pulmonary capillary bed, as in emphysema, the diffusing capacity falls. Therefore, the single-breath transfer factor of the lung for carbon monoxide (TLCO) is considered as the single best lung function measurement to assess severity of emphysema [8]. Furthermore, impaired TLCO is one of the strongest predictor of exercise capacity, points out oxygen desaturation during exercise, is highly related to hypoxaemia and poses a high risk for poor survival [11–13].

Respiratory muscle function has received considerable attention in patients with COPD as many studies have consistently shown that maximal static inspiratory pressures as well as oesophageal pressure are reduced [10]. These inspiratory muscles are faced to an increased elastic and resistive load in COPD, and the mismatch between the demand for respiratory muscle work and the capacity to meet that demand may partly explain common symptoms in COPD patients as dyspnea, hypercapnia and reduced tolerance to physical exercise [10, 14]. Arterial blood gas measurement is recommended in COPD patients to rule out significant hypoxemia or hypercapnia, particularly in patients with more severe disease [1].

These lung function measurements offer complementary information but cannot be used individually to accurately predict exercise performance, dyspnea, mood and health status in individual patients with COPD [5]. Taking into account the heterogeneity of the disease and in an attempt to improve the organization of care for patients with COPD, identifying patient profiles or COPD subtypes by means of clustering analysis has received growing attention [15–17]. Whether and to what extent a combination of the abovementioned lung function attributes correlates better with patient-related outcomes and clinical traits such as comorbidities was part of our hypothesis. Therefore, we aimed to cluster patients with COPD based on solely lung function attributes, derived from post-bronchodilator spirometry, TLCO, whole-body plethysmography, mouth pressures and resting arterial blood gases. *A priori*, we hypothesized that distinct clusters will be identified showing a large heterogeneity in the combination of lung function attributes in patients with COPD. Moreover, it is hypothesized that significant differences in exercise performance, health status and clinical traits as dyspnea and exacerbations will be found between these pathophysiological clusters, with still a substantial degree of heterogeneity within each of these clusters.

## Material and methods

### Study design

The current analysis used the data from the Chance Study: an observational, prospective, single-centre study about COPD, health status and cardiovascular comorbidities [18]. This study was approved by the Medical Ethical Committee of the Maastricht University Medical Centre+ (METC 11-3-070) and is registered at http://www.trialregister.nl (NTR 3416) (E-mail: secretariat.metc@mumc.nl).

### Study sample

Patients with clinically stable COPD [1] who were referred by a chest physician for a comprehensive pulmonary rehabilitation program at CIRO (Horn, the Netherlands) were eligible to participate. All patients gave written informed consent.

### Measurements

During a 3-day assessment, attributes related to COPD (including lung function), exercise performance, dyspnea, mood and health status were assessed.

#### Lung function

Post-bronchodilator spirometry was performed to assess forced expiratory volume in 1 second (FEV1) and forced vital capacity (FVC). Spirometry was measured with Masterlab® (Jaeger, Würzburg, Germany) following ATS/ERS guidelines [19]. Values are expressed as percentage of predicted according the Global Lung Function Initiative [20]. Total Lung Capacity (TLC), Residual Volume (RV) and Intra Thoracic Gas Volume (ITGV) were determined through body-plethysmography (Masterlab® Jaeger, Würzburg, Germany) following the quality control guidelines [21]. Values are expressed as a percentage of the European Coal and Steel Community predicted values [22]. TLCO was measured following the standard of the single-breath determination of carbon monoxide [23] and expressed in the reference values of Cotes and colleagues [24]. Additionally, TLCO per unit alveolar volume (KCO) was calculated. Maximal static inspiratory (MIP) and expiratory mouth pressures (MEP) were assessed according to ATS/ERS guidelines [25] and expressed in the reference values according to Black and Hyatt [26]. Resting arterial partial pressure of oxygen (PaO2), carbon dioxide (PaCO2) and oxygen saturation were measured (GEM4000, Instrumentation Laboratory, Peachtree City, USA). Patients with long term oxygen therapy (LTOT) continued oxygen supply during the procedure. All lung function measurements were performed by certified and experienced respiratory technicians.

#### Clinical, functional and health status characteristics

As described earlier [18], smoking history, number of exacerbations and hospitalizations for COPD in the previous twelve months, LTOT, self-reported comorbidities using the Charlson Comorbidity Index (CCI) [27], the degree of dyspnea using the modified Medical Research Council (mMRC) scale [28] and disease-specific health status using the COPD Assessment Test (CAT) [29], the Clinical COPD Questionnaire (CCQ) [30], and the COPD-specific version of the St George's Respiratory Questionnaire (SGRQ-C) [31] were assessed. Anxiety and depression were measured by the Hospital Anxiety and Depression Scale (HADS) [32]. Fat-free mass (FFM) was assessed using dual-energy X-ray absorptiometry (Lunar Prodigy system, GE Healthcare, Madison, WI, USA) and FFM was divided by squared height to obtain the FFM-index (FFMI). Low FFMI is defined as an FFMI below 16 kg/m^2^ for men and 15kg/m^2^ for women [33]. Exercise performance was assessed by a 6 minute walk test (6MWT) and by a symptom limited cardiopulmonary exercise test (CPET) using an electrically, braked cycle ergometer (Carefusion, Houten, the Netherlands) including the measurement of maximal oxygen uptake (Peak VO2 ml/min) and maximal work rate in Watts (Peak work rate). Furthermore, a submaximal exercise test at 75% of the peak work rate (CWRT) was performed. Isokinetic quadriceps muscle strength and endurance were measured using a Biodex (Biodex Medical Systems, Inc., New York, USA).

#### GOLD classification

Patients with COPD were classified as GOLD I to IV, and GOLD A to D, according the latest GOLD guideline [1].

### Statistics

All statistical analyses were performed using Viscovery Profiler 7.1 by Viscovery Software GmbH (www.viscovery.net; Vienna, Austria). Selforganizing maps (SOMs, also referred to as Kohonen maps) were used to create an ordered representation of the selected attributes. The SOM method can be viewed as a nonparametric regression technique that converts multidimensional data spaces into lower dimensional abstractions. A SOM generates a nonlinear representation of the data distribution and allows the user to identify homogeneous data groups visually. Patients have been ordered by their overall similarity concerning the lung function variables FEV1, % predicted; FEV1/FVC, %; FVC, % predicted; PEF, % predicted; ITGV, % predicted; RV, % predicted; TLC, % predicted; TLCO, % predicted; KCO, % predicted; MIP, % predicted; MEP, % predicted and arterial blood gases (PaO2, PaCO2) as well as SaO2, % and to a small extent the absolute measures of FEV1; FVC; PEF; ITGV; RV; TLC; TLCO; KCO; TLCHe; VIN; TA; MIP; and MEP measured during pre-rehabilitation assessment. Based on the created SOM model, clusters have been generated using the SOM-Ward Cluster algorithm of Viscovery, a hybrid algorithm that applies the classical hierarchical method of Ward on top of the SOM topology. Summary variables on clinical characteristics for the study sample and for each cluster are presented as mean + standard deviation for quantitative variables, and percentage for discrete variables. Viscovery automatically identified for each cluster all patient characteristics that differ significantly from the average of the whole study sample of 518 patients using the integrated two-sided t test with a confidence of 95%.

## Results

### Characteristics of the whole sample

Table 1 summarizes the characteristics of the whole sample of 518 patients. As a group, patients demonstrated marked airflow limitation and static hyperinflation. For the total group, TLCO was reduced with normal mean arterial blood gas values. Furthermore, patients generally had a normal body composition, MIP and MEP within normal ranges, an impaired exercise performance, deconditioned quadriceps muscles, and a poor health status. 24% of the patients used LTOT. The mean number of exacerbations as well as hospitalizations in the last year was on average 2.2 and 0.9. The majority of these patients was classified as GOLD D. Female COPD patients were younger, more hyperinflated and had worse gas exchange parameters than the male patients. Furtermore, higher symptoms of anxiety were seen in women compared to men.

**Table 1:** Lung function, clinical, functional and health status characteristics of the whole sample.

Characteristic	Whole sample N = 518	Female N = 230	Male N = 288	p-value parametric	p-value non-parametric
Women, %	44				
Age, years	64.1 (9.1)	62.5 (8.9)	65.4 (9.1)	< 0.001	< 0.001
FEV1, % predicted	48.6 (20)	49.1 (19)	48.2 (20)	0.628	0.448
FEV1/FVC, %	37.5 (12.2)	38.3 (11.8)	36.9 (12.6)	0.182	0.114
ITGV, % predicted	148.6 (35.9)	152.6 (33.9)	145.6 (37.0)	0.033	0.029
RV, % predicted	161 (50.7)	168.4 (48.2)	155.4 (51.9)	0.005	0.002
TLC, % predicted	117.1 (17.5)	122.4 (16.5)	113.1 (17.2)	< 0.001	< 0.001
TLCO, % predicted	49 (17)	47.8 (15.5)	50.5 (18.3)	0.082	0.094
KCO, % predicted	64 (21.9)	59.9 (19.3)	67.1 (23.2)	< 0.001	< 0.001
MIP, % predicted	78.5 (23.3)	87.0 (25.0)	71.7 (19.3)	< 0.001	< 0.001
MEP, % predicted	63.2 (20.4)	68.1 (22.2)	59.0 (17.7)	< 0.001	< 0.001
PaCO2, kPa	5.3 (0.9)	5.4 (0.9)	5.2 (0.9)	0.021	0.014
PaO2, kPa	9.5 (1.5)	9.5 (1.4)	9.6 (1.5)	0.583	0.569
SaO2, %	93.9 (3.2)	93.8 (3.2)	93.9 (3.2)	0.530	0.237
Exacerbations <1 year, n	2.2 (1.8)	2.4 (1.8)	2.1 (1.8)	0.054	0.041
Hospitalizations <1 year, n	0.9 (1.3)	0.9 (1.3)	0.9 (1.3)	0.811	0.894
mMRC dyspnea grade	2.4 (1.0)	2.4 (1.0)	2.4 (1.1)	0.782	0.890
LTOT use, %	24.1	25.7	22.9		0.470
Pack years	42.4 (23.6)	41.0 (22.7)	43.5 (24.2)	0.233	0.413
CCI, points	1.6 (1.0)	1.5 (0.8)	1.8 (1.1)	0.001	0.002
Patients with GOLD I / II /III / IV, %	7 / 36 / 37 / 20	6/38/39/17	8/34/35/22		0.354
Patients with GOLD A / B / C / D, %	3/20/5/72	2/17/3/79	4/23/7/67		0.010
6MWD, m	424 (124.4)	412.9 (118.9)	432.9 (128.1)	0.071	0.068
6MWD, % predicted	67.1 (18)	70.1 (17.3)	64.7 (18.1)	0.001	< 0.001
Peak Vo2, % predicted	66.2 (30.4)	85.3 (32.8)	51.5 (17.7)	< 0.001	< 0.001
Peak work rate, % predicted	55.5 (27.4)	70.1 (29.7)	44.1 (18.7)	< 0.001	< 0.001
CWRT, s	295.5 (218.7)	264.5 (177.4)	319.3 (243.4)	0.006	0.022
Quadriceps peak torque, % predicted	66.2 (18.9)	65.8 (18.6)	66.6 (19.1)	0.667	0.438
BMI, kg/m2	26.2 (5.8)	25.9 (5.8)	26.5 (5.8)	0.196	0.147
FFMI, kg/m2	17.2 (2.6)	15.6 (2.1)	18.4 (2.3)	< 0.001	< 0.001
HADS-A, points	7.8 (4.5)	8.7 (4.8)	7.1 (4.1)	< 0.001	< 0.001
HADS-D, points	7.5 (4.3)	7.9 (4.7)	7.3 (4.0)	0.106	0.202
SGRQ, total score, points	61.1 (17.4)	62.5 (16.8)	60.0 (17.8)	0.105	0.085
CAT, total score, points	21.5 (6.6)	22.7 (6.1)	20.6 (6.9)	< 0.001	0.001
CCQ, total score, points	2.6 (1.0)	2.7 (1.0)	2.6 (1.0)	0.102	0.080

Data are presented as mean (SD). FEV1: forced expiratory volume in 1 s; FVC: forced vital capacity; ITGV: intra thoracic gas volume; RV: residual volume; TLC: total lung capacity; KCO: the single-breath transfer factor of the lung for carbon monoxide (TLCO) per unit alveolar volume; MIP: maximal static inspiratory mouth pressure and MEP: maximal static expiratory mouth pressure; PaCO2: arterial partial pressure of oxygen and PaCO2: arterial partial pressure of carbon dioxide; SaO2: arterial oxygen saturation; mMRC: modified medical research council; LTOT: long-term oxygen therapy; CCI: Charlson Comorbidity index; 6MWD: 6-minute walking distance; VO2: oxygen uptake; CWRT: constant work-rate test; BMI: body mass index; FFMI: fat-free mass index; HADS-A: hospital anxiety and depression scale, anxiety scores; HADS-D: hospital anxiety and depression scale, depression scores; SGRQ: St. George’s respiratory questionnaire; CAT: COPD assessment test; CCQ: clinical COPD questionnaire.

### The lung function clusters

**Fig 1:**
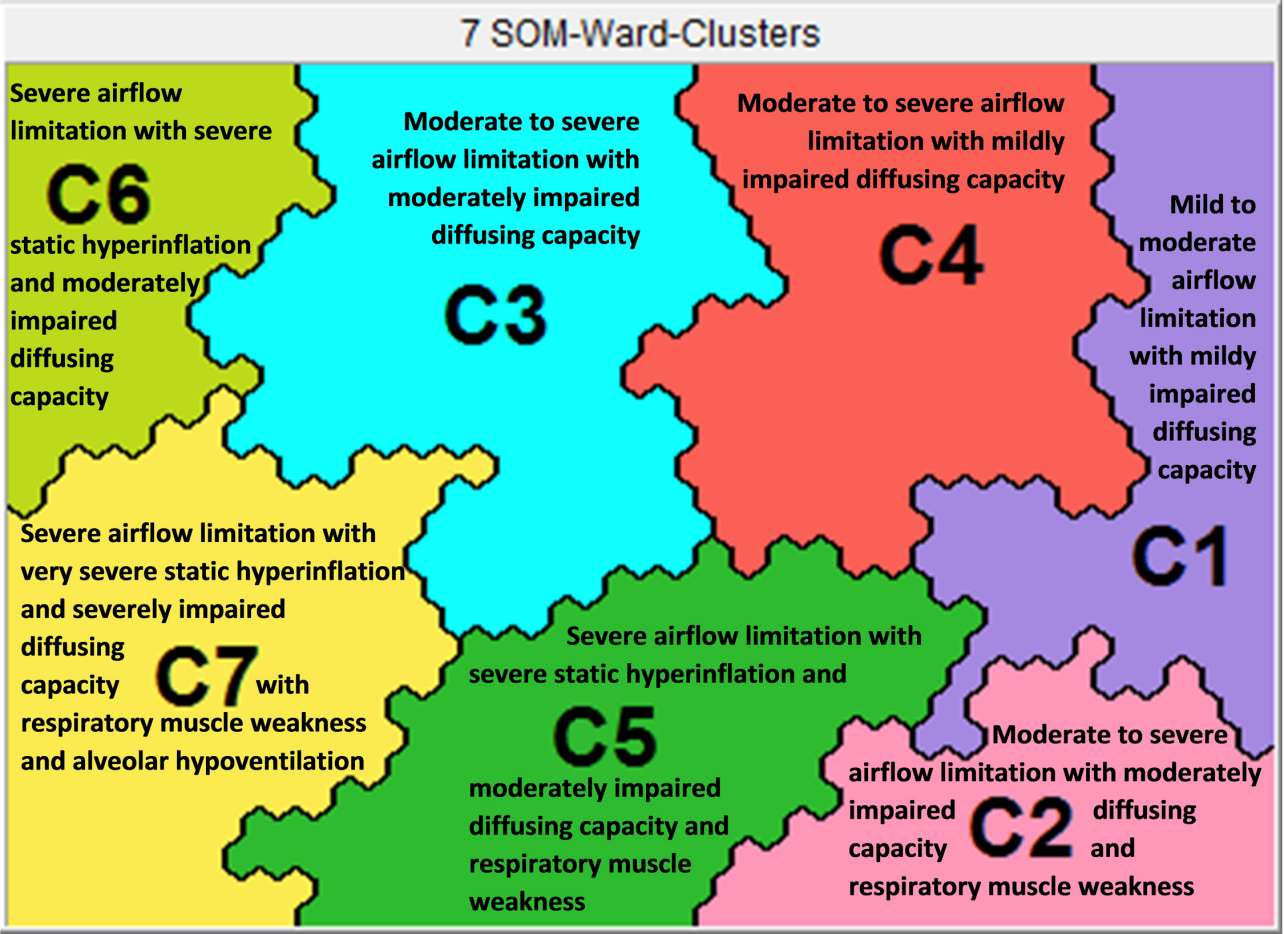
Heterogeneity of lung function impairment in COPD.

**Fig 2:**
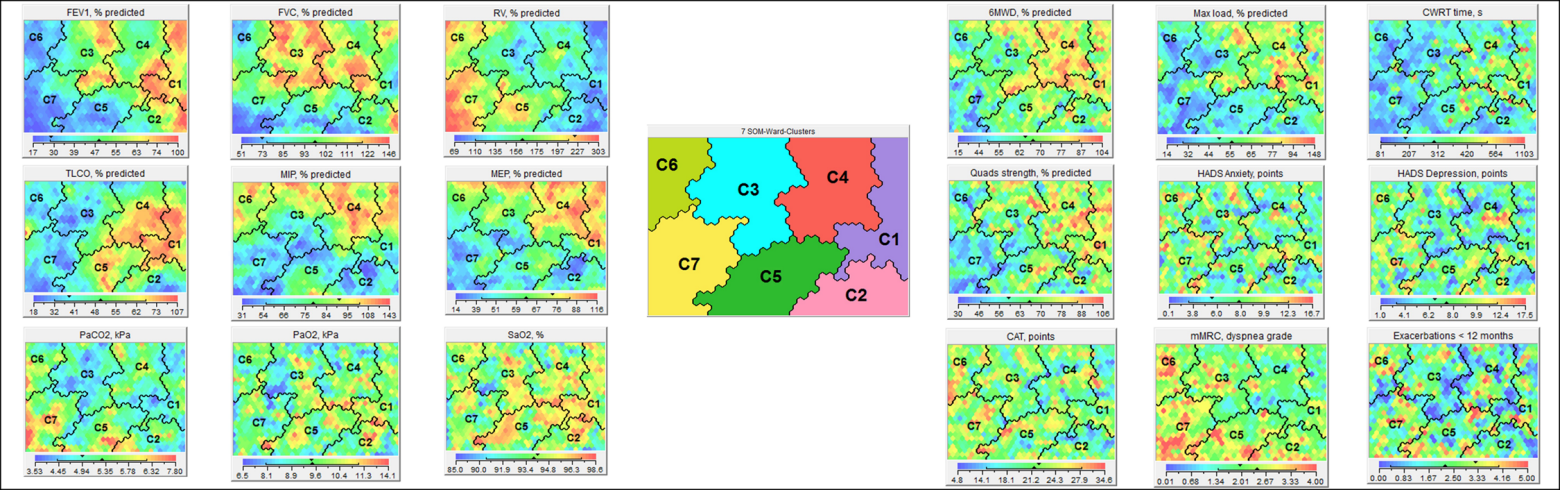
The lung function clusters and the related functional and health status characteristics. The seven lung function clusters in chronic obstructive pulmonary disease (COPD) and the related functional and health status characteristics. When looking at the different lung function, functional characteristics and health status, subjects “raise a red flag” if the attribute is relatively high within this sample, present “a green flag” if the clinical attribute is moderate, and present “a blue flag” when the clinical attribute is relatively low within this sample. In this way the maps can be interpreted. The Viscovery program placed all subjects on a specific position on the map based on their profile of a comprehensive lung function assessment. The more subjects resemble in terms of their lung function the closer they are on the map. Contrarily, the more they differ the further they are away from each other. By drawing lines on the map, the Viscovery program could identify seven different clusters of patients with COPD with a significant different respiratory physiome (95% confidence interval).

SOMs resulted in seven clusters with significantly different lung function profiles (Fig 1). As shown in Table 2 and Fig 2, a clear dichotomy is depicted for the spirometric (higher in clusters 1 to 4; lower in clusters 5 to 7) and static lung volumes (higher in clusters 5 to 7; lower in clusters 1, 2, and 4).

**Table 2:** Lung function attributes of the seven clusters.

Lung function attribute	Cluster 1N = 75	Cluster 2N = 61	Cluster 3N = 89	Cluster 4N = 79	Cluster 5N = 66	Cluster 6N = 61	Cluster 7N = 87
*Post-bronchodilator spirometry*							
FEV1, % predicted	71 (12)† ‡ ¡ § ¶ ị	59 (21)§ ¶ ị	58 (15)§ ¶ ị	55 (12)§ ¶ ị	37 (12)¶ ị	31 (6)	29 (10)
FEV1/FVC, %	55 (7)† ‡ ¡ § ¶ ị	47 (10)‡ ¡ § ¶ ị	37 (10)§ ¶ ị	39 (7)§ ¶ ị	32 (10)¶ ị	28 (5)	27 (6)
FVC, % predicted	100 (16)† ‡ ¡ § ¶ ị	92 (22)‡ ¡ § ị	121 (14)¡ § ¶ ị	108 (14)§ ¶ ị	86 (13)	88 (14)ị	81 (22)
PEF, % predicted	94 (15)† ‡ ¡ § ¶ ị	73 (25)§ ¶ ị	69 (20)§ ¶ ị	74 (17)§ ¶ ị	52 (13)¶ ị	46 (10)	43 (15)
*Whole-body plethysmography*							
ITGV, % predicted	105 (17)† ‡ ¡ § ¶ ị	115 (20)‡ ¡ § ¶ ị	155 (26)¡ ¶ ị	136 (18)§ ¶ ị	158 (19)¶ ị	179 (21)ị	192 (26)
RV, % predicted	109 (22)‡ ¡ § ¶ ị	115 (25)‡ ¡ § ¶ ị	154 (35)¡ § ¶ ị	144 (22)§ ¶ ị	178 (28)¶ ị	207 (36)ị	224 (44)
TLC, % predicted	99 (11)‡ ¡ § ¶ ị	95 (13)‡ ¡ § ¶ ị	127 (14)¡ §	118 (9)¶ ị	117 (10)¶ ị	131 (12)	131 (13)
*Carbon monoxide diffusion capacity*							
TLCO, % predicted	65 (16)† ‡ ¡ § ¶ ị	45 (15)¡ § ị	42 (12)¡ § ị	60 (16)§ ¶ ị	54 (14)¶ ị	42 (15)ị	36 (9)
KCO, % predicted	88 (22)† ‡ ¡ § ¶ ị	67 (23)‡ § ¶ ị	48 (13)¡ § ¶	71 (15)¶ ị	76 (18)¶ ị	52 (11)	49 (11)
*Mouth pressures*							
MIP, % predicted	89 (21)† ¡ § ị	59 (16)‡ ¡ § ¶	83 (22)¡ § ¶ ị	97 (19)§ ị	68 (17)¶ ị	94 (12)ị	58 (14)
MEP, % predicted	78 (18)† ‡ ¡ § ¶ ị	48 (16)‡ ¡ ¶	67 (17)¡ § ị	84 (14)§ ¶ ị	53 (12)¶ ị	65 (14)ị	44 (13)
*Arterial blood gases*							
PaCO2, kPa	4.9 (0.6)† ¡ § ¶ ị	5.2 (0.8)‡ § ị	4.8 (0.5)¡ § ¶ ị	5.2 (0.6) § ¶ ị	5.5 (0.8)ị	5.4 (0.7)ị	6.0 (1.2)
PaO2, kPa	9.9 (1.4)‡ ị	9.6 (2.1)	9.4 (1.5)	9.6 (1.5)	9.8 (1.4)ị	9.6 (1.1)	9.2 (1.4)
SaO2, %	94.8 (2.3)† ‡ ị	93.6 (3.9)	93.7 (3.0)	94.0 (3.2)	94.3 (3.0)ị	94.1 (2.1)ị	92.9 (4.1)

See Table 1 legend for expansion of abbreviations.

Purple: A significantly higher value compared to the value of the remaining six clusters together (*P*<0.05)

Green: A significantly lower value compared to the value of the remaining six clusters together (*P*<0.05)

†: p<0.01 versus cluster 2;

‡: p<0.01 versus cluster 3;

¡: p<0.01 versus cluster 4;

§: p<0.01 versus cluster 5;

¶: p<0.01 versus cluster 6;

ị: p<0.01 versus cluster 7.

Cluster 1 had a significantly lower degree of airflow limitation, absence of static hyperinflation, and a higher diffusing capacity compared to the other clusters. Clusters 2 to 4 had similar degree of airflow limitation, but showed significant differences in static lung volumes (Cluster 3 > Cluster 4 > Cluster 2). Cluster 5 had significantly higher spirometric lung volumes compared to Clusters 6 and 7. Static lung volumes were significantly different between Clusters 5 to 7 (Cluster 7 > Cluster 6 > Cluster 5). A differential pattern occurred for TLCO (higher in Clusters 1, 4 and 5; lower in Clusters 3, 6, and 7); mouth pressures (higher in Clusters 1, 3, 4, and 6; lower in Clusters 2, 5, and 7). Arterial blood gas values were within normal ranges in all clusters except of cluster 7.

### Functional and health status characteristics of clusters

Table 3 and Fig 2 show the functional characteristics and health status related to the seven different lung function profiles. Clusters 1 and 4 generally had the best scores for attributes related to physical fitness (i.e., 6MWD, peak VO2, peak work rate, and quadriceps muscle function) and health status questionnaires (SGRQ, CAT, and CCQ), while this was worst for Clusters 6 and 7.

**Table 3:** Functional and health status characteristics of the seven lung function clusters.

	Cluster 1N = 75	Cluster 2N = 61	Cluster 3N = 89	Cluster 4N = 79	Cluster 5N = 66	Cluster 6N = 61	Cluster 7N = 87
6MWD, m	466 (118)† § ¶ ị	430 (131)‡ ¡ ị	445 (115)¡ ¶ ị	495 (94)§ ¶ ị	413 (100)ị	400 (112)ị	340 (131)
6MWD, % predicted	76 (16)† ‡ § ¶ ị	64 (18)‡ ¡ ị	70 (16)¡ § ¶ ị	80 (12)§ ¶ ị	64 (13)ị	63 (16)ị	53 (19)
Peak Vo2, % predicted	81 (36)† ‡ § ¶ ị	55 (16)‡ ¡	68 (30)¡ § ị	82 (29)§ ¶ ị	55 (21)	58 (22)ị	47 (26)
Peak work rate, % predicted	75 (32)† ‡ § ¶ ị	48 (19)‡ ¡ ị	60 (26)¡ § ¶ ị	73 (26)§ ¶ ị	43 (16)ị	47 (18)ị	36 (21)
CWRT, s	356 (225)‡ ¶ ị	307 (297)	266 (173)¡	353 (221)¶ ị	293 (216)	247 (136)	242 (222)
Quadriceps peak torque, % predicted	79 (15)† ‡ § ¶ ị	63 (20)‡ ¡ ị	69 (17)¡ ¶ ị	76 (19)§ ¶ ị	64 (16)ị	62 (17)ị	51 (14)
BMI, kg/m2	31 (6)† ‡ ¡ § ¶ ị	27 (5)‡ ¶ ị	25 (5)¡ ị	28 (6)§ ¶ ị	26 (5)ị	24 (5)ị	22 (5)
FFMI, kg/m2	19 (3)† ‡ ¡ § ¶ ị	18 (2)‡ ¶ ị	17 (2)¡ § ¶ ị	18 (3)¶ ị	18 (3)¶ ị	16 (2)	16 (2)
mMRC dyspnea grade	2.0 (1.0)† § ¶ ị	2.6 (1.1)‡ ¡ ị	2.1 (1.0)§ ¶ ị	2.0 (1.0)§ ¶ ị	2.7 (0.9)	2.8 (0.9)	2.9 (1.0)
SGRQ, points	57 (21)§ ¶ ị	61 (18)¡ § ¶ ị	58 (17)§ ¶ ị	53 (15)§ ¶ ị	67 (15)	67 (15)	67 (16)
CAT, points	20 (7)§ ¶ ị	20 (7)§ ¶ ị	21 (6)¶	21 (7)§ ¶	23 (6)	24 (6)	23 (6)
CCQ, points	2.3 (1.1)§ ¶ ị	2.6 (1.2)ị	2.5 (0.9)§ ¶ ị	2.3 (0.9)§ ¶ ị	2.8 (0.9)	3.0 (1.0)	3.0 (0.9)

See Table 1 legend for expansion of abbreviations.

Purple: A significantly higher value compared to the value of the remaining six clusters together (*P*<0.05)

Green: A significantly lower value compared to the value of the remaining six clusters together (*P*<0.05)

†: p<0.01 versus cluster 2;

‡: p<0.01 versus cluster 3;

¡: p<0.01 versus cluster 4;

§: p<0.01 versus cluster 5;

¶: p<0.01 versus cluster 6;

ị: p<0.01 versus cluster 7.

### Clinical characteristics and GOLD classification of clusters

The clinical characteristics of the seven clusters are summarized in Table 4. Clusters 2 and 5 were older and had a higher proportion of men while Cluster 6 had a higher proportion of women, as did Cluster 3, with a younger mean age. Exacerbations in the last 12 months were higher in Clusters 5 and 7, while this was lower in Cluster 3. A similar pattern was observed for hospitalizations (higher in Cluster 7; lower in Clusters 3 and 4). The proportion of patients using long-term oxygen was higher in Cluster 7, and lower in Clusters 1 and 4. Clusters 1 and 2 had higher scores on the Charlson comorbidity index, which was lower in Cluster 4. Clusters 6 and 7 had a higher mean dyspnea score. Remarkably, about one quarter of the patients in cluster 1, 3 and 4 were classified as GOLD B and about half of the patients in cluster 1 to 4 were COPD GOLD D patients. Otherwise, practically all patients of clusters 5 to 7 were classified as GOLD D.

**Table 4:** Clinical characteristics and GOLD classification of the seven lung function clusters.

	Cluster 1N = 75	Cluster 2N = 61	Cluster 3N = 89	Cluster 4N = 79	Cluster 5N = 66	Cluster 6N = 61	Cluster 7N = 87
Women, %	41.3† § ¶	19.7‡ ¡ ¶ ị	53.9§ ¶	53.2§ ¶	21.2¶ ị	77ị	41.4
Age, years	64 (10)† ¶	69 (9)‡ ¡ ¶ ị	64 (9)¶	63 (9)§ ¶	67 (9)¶ ị	59 (8)ị	63 (8)
Exacerbations <1 year, n	2.1 (2.0)	1.8 (1.7)§ ¶ ị	1.8 (1.6)§ ¶ ị	2.0 (1.8)§ ị	2.6 (1.8)	2.5 (1.8)	2.6 (1.8)
Hospitalizations <1 year, n	0.7 (1.1)¡ ị	1.1 (1.3)‡ ¡	0.5 (0.9)§ ¶ ị	0.4 (0.8)§ ¶ ị	1.1 (1.4)	1.0 (1.4)	1.4 (1.6)
LTOT use, %	8.0† ‡ § ¶ ị	32.8¡	19.1ị	13.9§ ¶ ị	28.8	31.1	37.9
Pack years	44 (26)	41 (25)	42 (20)	44 (31)	44 (24)	39 (14)	41 (21)
CCI, points	2.0 (1.1)‡ ¡ § ¶ ị	2.1 (1.5)‡ ¡ § ¶ ị	1.5 (0.8)	1.4 (0.8)	1.5 (0.9)	1.6 (1.0)	1.5 (0.8)
HADS-A, points	7.8 (4.2)	7.1 (4.6)	7.7 (4.7)	7.3 (3.5)	7.6 (4.3)	8.5 (5.0)	8.6 (5.0)
HADS-D, points	7.2 (4.3)	7.6 (4.6)	7.0 (4.4)	6.9 (4.0)	7.9 (3.9)	7.9 (4.2)	8.3 (4.8)
Patients with GOLD 1, %	28.0‡ ¡ § ¶ ị	14.8¡ § ¶ ị	5.6ị	3.8	0	0	0
Patients with GOLD 2, %	66.7† § ¶ ị	45.9§ ¶ ị	59.6§ ¶ ị	53.2§ ¶ ị	15.2¶ ị	0	2.3
Patients with GOLD 3, %	5.3† ‡ ¡ § ¶ ị	39.3¶	33.7§ ¶	43.0	53.0ị	59ị	32.2
Patients with GOLD 4, %	0§ ¶ ị	0§ ¶ ị	1.1§ ¶ ị	0§ ¶ ị	31.8ị	41ị	65.5
Patients with GOLD A, %	17.3§ ¶ ị	11.5§ ¶ ị	12.4§ ¶ ị	8.9¶ ị	1.5	0	0
Patients with GOLD B, %	25.3§ ¶ ị	14.8§ ¶ ị	22.5§ ¶ ị	21.5§ ¶ ị	3.0	0	0
Patients with GOLD C, %	10.7	9.8	13.8	19.0§ ị	6.3	8.3	6.9
Patients with GOLD D, %	46.7† § ¶ ị	63.9§ ¶ ị	50.6§ ¶ ị	50.6§ ¶ ị	88.9	91.7	93.1

See Table 1 legend for expansion of abbreviations.

Purple: A significantly higher value compared to the value of the remaining six clusters together (*P*<0.05)

Green: A significantly lower value compared to the value of the remaining six clusters together (*P*<0.05)

†: p<0.01 versus cluster 2;

‡: p<0.01 versus cluster 3;

¡: p<0.01 versus cluster 4;

§: p<0.01 versus cluster 5;

¶: p<0.01 versus cluster 6;

ị: p<0.01 versus cluster 7.

Mean scores for anxiety and depression were not significantly different between Clusters. As expected from the lung function attributes, clear differences were observed in the GOLD classification per Cluster.

## Discussion

This is the first study clustering patients with mild to very severe COPD based on a comprehensive lung function assessment, including post-bronchodilator spirometry, TLCO, whole-body plethysmography, mouth pressures, and arterial blood gases. Seven clusters were identified, with distinct patterns of lung function impairment demonstrating the complexity and heterogeneity of pathophysiological changes in the respiratory system of COPD patients referred for pulmonary rehabilitation. Our data indicate that simple classification of COPD patients based on spirometry and health status or breathlessness underestimates this heterogeneity in respiratory impairment as well as the identifiable treatable traits in an integrated and individualized management plan for COPD.Significant differences were found in gender distribution, age, exacerbations/hospitalizations, comorbidities, physical fitness, and health status between clusters, only partially related to the degree of lung function impairment. Symptoms of anxiety and depression were comparable between the seven clusters. Large heterogeneity for the abovementioned functional and clinical characteristics still existed within each cluster. Therefore, clustering of lung function attributes does still not allow to accurately determine functional characteristics and health status in individual patients with COPD. These findings emphasize the need of a comprehensive assessment of patients with COPD to gain insight in the different respiratory and systemic treatable traits of the disease in the individual patient in order to understand the true burden of the disease.

Clusters with the best functional performance and health status (clusters 1 and 4) had the lowest extent of airflow limitation, alveolo-capillary membrane damage, the best respiratory muscle function and absent or mild static hyperinflation.

Although GOLD guidelines mention that gas exchange abnormalities result in hypoxemia and hypercapnia, no further recommendation is provided about TLCO measurement to assess the severity, complexity and heterogeneity of COPD [1]. The current study suggests that quantitative assessment of gas transfer in the lungs offers additional information of respiratory involvement in COPD as part of a standard lung function test. Our study confirms previous findings that reduced TLCO along with airflow limitation identifies those patients with significant more symptoms [11]. Intriguingly, both clusters with female predominance (clusters 3 and 6) had manifested impaired TLCO.

Lung hyperinflation, the ultimate consequence of expiratory airflow limitation, importantly contributes to the degree of dyspnea, exercise limitation, impaired left ventricular filling and increased cardiovascular mortality associated with the disease [9]. Our study confirms that clusters with the highest level of static hyperinflation had the worst health and functional status and the highest exacerbation and hospitalization rates, indicating the impact of respiratory mechanics on COPD related disease burden.

Respiratory muscle function in COPD has received considerable attention in the last decades. Generally, a reduction of MIP is reported in COPD patients [10]. Intriguingly, our analysis demonstrated a normal respiratory muscle function despite presence of static hyperinflation in cluster 6 while cluster 2 manifested a reduced MIP and MEP despite absence of hyperinflation and absence of nutritional depletion. The same cluster also had manifested lower quadriceps muscle dysfunction and reduced peak exercise performance suggesting underlying intrinsic muscular abnormalities. Stratifying COPD patients based on this heterogeneity of respiratory muscle dysfunction and underlying factors may offer new perspectives for respiratory muscle training as part of an integrated management strategy in these patients.

Interesting are the gender differences between the different clusters with a high prevalence of females in clusters 3 and 6 and a relatively low number of females in clusters 2 and 5. Clusters 3 and 6 had the most impaired diffusing capacity with normal respiratory muscle strength, opposite to the lung function changes in both male predominant clusters. Furthermore, marked age differences exist between cluster 3 and 6. These data are confirming previous findings of a female predominance in severe, early onset COPD [34]. Our data also support the findings of Pinto et al, based on a systematic review of clinican phenotypes in COPD [35]. They describe one phenotype of younger COPD patients with very severe respiratory disease, a low probability of cardiovascular comorbidities, a high prevalence of poor nutrional status and poor health status with poor longitudinal outcomes [35]. Severily impaired diffusing capacity as illustrated in our analysis seems to be an important pathophysiological characteristic in these patients and offers new therapeutic avenues to treat the disease more aggressively at younger age. Although symptoms for anxiety and depression were comparable between the seven clusters, the presence of higher levels of anxiety and depression in women with COPD may also impact the burden of the disease in these patients [35]. Also cluster 1 in our study clearly illustrates the limitations of this pathophysiological approach: despite mild impairment of lung function, this cluster of COPD patients had a high disease burden as reflected by worse health status, experienced dyspnea and high rate of even severe exacerbations. This cluster emphasizes the fact that the daily burden of COPD is influenced by factors beyond the lungs and that the presence of comorbidities may explain the impact on health status and functional status [36, 37]

Combined with reported gender differences in clinical presentation, different patterns of comorbidities as well as in response to therapeutic modalities, gender-specific treatment and management strategies must be considered in current medical practice.

Our study clearly illustrates that a variety of pathophysiological respiratory impairments can result in comparable levels of functional impairment, advocating the need for thorough assessment of the individual patient to understand the burden of disease and to select more individualized and targeted intervention strategies [38]. Recently, a label-free precision medicine approach for management of chronic airway diseases has been proposed based on identification of treatable respiratory, extra-pulmonary and behavior/life style traits [39].

Considering the outcomes of summative outcome measurements as exercise performance tests as well as health status measurements, our study clearly illustrates that a variety of pathophysiological respiratory impairments can result in comparable levels of functional impairment, advocating the need for thorough assessment of every patient to understand the level of physical functioning and to select more individualized and targeted intervention strategies [39]. Our data properly emphasizes that selection or restriction of pulmonary rehabilitation cannot be based on one single lung function characteristic as formulated in international recommendations for management of stable COPD [1]. Such guidelines completely ignore that patients greatly differ in terms of how this complex disease can affect their lives.

### Methodological considerations

The current study has several strengths: 1) a total of 518 well-characterized patients with COPD were analyzed, including patients with GOLD stages 1 to 4, and A to D; 2) the SOMs allowed us to visualize the ratio between the various lung function attributes and attributes related to clinical and functional characteristics and health status, which extends our current insights. However, some limitations need to be considered. First, the current sample contained COPD patients who were referred by chest physicians to a comprehensive pulmonary rehabilitation program. Moreover, the majority of these COPD patients were classified in group D. Therefore, the current findings need to be corroborated in different COPD samples. Second, follow-up studies will also be needed to validate our identified clusters in other cohorts as well as transition of clusters over time [17]. Indeed, four different clusters of lung function trajectories were recently identified in smokers with and without COPD [40]. Third, given the cross-sectional nature of the clusters, the relevance in terms of longterm outcomes needs also validation in prospective studies. Fourth, only resting hyperinflation was used in the current approach. Dynamic hyperinflation as part of the pathophysiological attributes used for clustering needs to be evaluated. Then again, it is known that the extent of dynamic hyperinflation inversely varies with the level of resting hyperinflation in patients with COPD [41] assuming that current findings will not be importantly modified. Fifth, diffusing capacity measurements are used as a surrogate marker of alveolar tissue loss related to emphysema [42]. Future studies need to consider quantification of the degree and distribution of emphysema using advanced imaging procedures as computed tomography. Finally, pulmonary hemodynamics will complement the COPD related changes in the respiratory system.

## Conclusion

To conclude, patients with COPD can be clustered based on a comprehensive lung function assessment. The current findings clearly show that the FEV1 is not a *pars pro toto* for the respiratory impairment in patients with COPD. Moreover, FEV1 or any other single lung function parameter cannot be used to predict the functional characteristics and health status. Our study emphasizes the contributing role of different pulmonary function tests and that different pathophysiological mechanisms lead to a comparable level of functional deterioration. So, a comprehensive assessment, including detection of altered pathophysiological mechanisms, should become essential to understand the personal burden in patients with COPD, to identify treatable traits and to understand the heterogeneity of structure-function relationships in COPD patients.
